# Power Distance Belief and Workplace Communication: The Mediating Role of Fear of Authority

**DOI:** 10.3390/ijerph19052932

**Published:** 2022-03-02

**Authors:** Yuwan Dai, Hao Li, Wenting Xie, Tianyi Deng

**Affiliations:** 1 Beijing Key Laboratory of Behavior and Mental Health, School of Psychological and Cognitive Sciences, Peking University, Beijing 100871, China; daiyuwan@pku.edu.cn; 2 Plateau Brain Science Research Center, Tibet University, Lhasa 850001, China; 3 School of Education, Tibet University, Lhasa 850001, China; gszyxwt@163.com; 4 Health Science Center, Peking University, Beijing 100871, China; 1710120119@pku.edu.cn

**Keywords:** power distance belief, fear of authority, workplace accidents, communication with superiors

## Abstract

Power distance is the degree of acceptance of unequal distribution of power in societies. In a high power distance context, the acceptance of inequality conflicts with the operation of modern organizations, which causes obstacles to workplace communication or even triggers workplace accidents due to ineffective communication. We conducted four studies (*N* = 1063) to explore the relations between and mechanisms of power distance belief and workplace communication. In Study 1, the participants with high power distance belief had ineffective workplace communication—specifically ineffective communication with superiors—but no difference in communication with subordinates and colleagues. We further focused on the mechanism underlying the relationship between power distance belief and communication with superiors. A questionnaire study (Study 2) was conducted in three stages over a three-month period, and an experimental study (Study 3) indicated that fear of authority mediated the negative effect of high power distance on communication with superiors. A cross-culture study (Study 4) re-tested the hypotheses among Chinese and U.S. participants. This research provides insight into the mechanisms that explain the relationship between power distance belief and workplace communication, indicating that fear of authority is significant. Organizations should pay attention to power distance belief and fear of authority, as they may lead to workplace accidents due to communication disasters.

## 1. Introduction

Workplace communication plays a crucial role in industrial organization with the development of economic globalization as well as the greater complexity of organizational structures. Cultural factors play an important role in workplace communication [1,2,3]. Individualism–collectivism affects communication styles directly and also has an indirect effect that is mediated through self-constructs and values [4,5]. As another important cultural factor, power distance belief has also been found to influence communication between teachers and students [6]. In our current research, we aimed to explore the relationship between power distance belief and workplace communication. Moreover, we proposed that fear of authority could account for the effect of power distance belief on workplace communication.

### 1.1. High Power Distance Leads to Inefficient Communication

Power distance belief refers to the degree to which individuals, groups, or societies accept the unequal distribution of power, state, or wealth in society or organizations [7]. Power distance belief is originally a homogeneous national value [7]; it is also a dispositional trait at the individual level [8,9,10]. Daniels and Greguras [11] indicated that low power distance countries embraces both high power distance belief individuals and low power distance belief individuals. The central characteristic of power distance is acceptance of inequality [7,11]. Specifically, in terms of attitude, individuals with high power distance belief are more likely to accept power inequalities in their organizations [12]. Accordingly, they would approve of hierarchy and hold the opinion that subordinates should trust and obey the superiors [13,14]. In terms of behavior, individuals with high power distance belief hold weaker trust in others [15], more feedback seeking from peers, less feedback seeking from superiors [16], and weaker innovation [17].

Effective communication is essential to the survival of all organizations [18,19]. Communication refers to a transaction whereby individuals generate meaning through the exchange of symbols [20]. Two central characteristics of communication are interaction and achieving consistency in the meaning of symbols [21]. Communication occurs every day in organizations. Effective communication contributes to the daily running, promotes organization benefits, and avoids workplace accidents [22,23,24]. Previous studies focused on improving personal communication skills (e.g., listening well, speaking clearly, inquiring skillfully) to enhance workplace communication [25]. Recent studies indicated that cultural factors play an important role in workplace communication [2,3].

Psychologists have a theoretical speculation on the link between power distance belief and effective communication at work [18]. Suter et al. [26] further conducted qualitative research and found that participants emphasize effective communication as a core competency for collaborative practice in interviews. Previous research provides pieces of experimental evidence for this link. On the one hand, individuals with high power distance in organizations interact less in the organization. Ji et al. [15] found that high power distance individuals are less likely to seek help. Varela and Premeaux [27] found that subordinates with high power distance tend to keep silent and are less willing to provide feedback. On the other hand, individuals with high power hold low communication openness, which hinders the exchange of information. Superiors with higher power distance belief tend to have an egocentric decision-making style [7], distrusting subordinates’ opinions [10]. Meanwhile, subordinates with high power distance belief tend to rely on superiors’ opinions for decision-making [7], tolerate supervisory mistreatment [28], and perform more upward ingratiatory behavior to please leaders even if it is against objective facts [29]. Hence, we proposed that high power distance belief is likely to inhibit people from communicating effectively. This is our first Hypothesis (H1):

**Hypothesis** **1** **(H1).** *High power distance belief is positively associated with more ineffective communication in the workplace.*

### 1.2. Fear of Authority Can Result in Inefficient Communication

Fear of authority is an emotional state in which individuals feel fearful of authority and the evaluation of authority [30]. Opposite to positive emotions such as admiration and respect, fear of authority is a negative emotion in valence and holds an avoidance motivation [31,32]. Emotionally, in addition to fear and anxiety, individuals may experience self-doubt, disgust, disappointment, and anger [33,34]. Behaviorally, fear elicits alienation, avoiding authority [35], obedience, conflict avoidance [36], and expectation of approval [37]. Fear of authority is elicited not only by authorities and experts but also by high social status, elderly people, superiors, and other people of higher social classes [38], and even people who dress as if “higher class” can cause those with low self-confidence to experience a fear of authority [39].

Fear of authority can decrease the effectiveness of communication. Emotional experiences such as fear and anxiety elicit avoidance behavior rather than approach behavior [40,41]. An avoidance behavior that seems ubiquitous in this regard is remaining silent [42,43]. Keeping silent results in less communication, discrete information sharing, and poor feedback. Furthermore, as in the well-known Milgram studies of obedience to authority, it is prevalent to obey authority even when doing so is problematic [44]. Fear of authority results in compliance with the authority’s orders without consideration. Fear of authority allows superiors to exert strong social pressure, which puts the individual in a situation of conformity, as in peer-pressure situations [45]. As a result, the individual tends to avoid responsibility and tends to follow orders without question. In addition, individuals with a high fear of authority tend to believe that authority naturally means correct and professional, which can result in cognitive bias. Accordingly, challenging the views of authority would be incorrect, and agreeing is a wise choice. Fear of authority can also lead individuals to change their own opinions to be consistent with the authority in terms of their thoughts, attitudes, and behaviors. The most frequently mentioned reason for remaining silent is the fear of being viewed or labeled negatively, and thus, damaging valued relationships [46]. In high fear of authority situations, individuals often have increased fear of negative evaluations and social anxiety, which weaken their self-esteem [47,48]. Seeking consistency with authority is a means of obtaining connections and improving self-esteem [49]. Even if individuals have doubts about their superiors’ decisions, they may be unwilling to express this doubt and even persuade themselves that the decision is correct to address the cognitive dissonance. Therefore, we proposed our second Hypothesis (H2):

**Hypothesis** **2** **(H2).** *Fear of authority is positively associated with more ineffective communication in the workplace.*

Fear of authority is an emotional experience that is influenced by personality [50], environmental context [35], and culture [10]. Power distance is an important cultural factor, and previous studies have found that power distance is associated with emotional expression and emotional experience [51]. For example, under high power distance conditions, people are more eager to be accepted by others [52]. In addition, Mondillon et al. [53] indicated that people with power tend to feel more anger and pride, and people without power feel more fear and sadness. People with high power distance belief are more likely to perceive their superiors as authoritarian [11,51] and feel less powerful, which may generate a higher sense of fear of authority. Therefore, we proposed our third Hypothesis (H3) and fourth Hypothesis (H4):

**Hypothesis** **3** **(H3).** *Power distance belief is positively associated with fear of authority.*

**Hypothesis** **4** **(H4).** *Fear of authority mediates the effect of power distance belief on workplace communication.*

**Figure 1:**
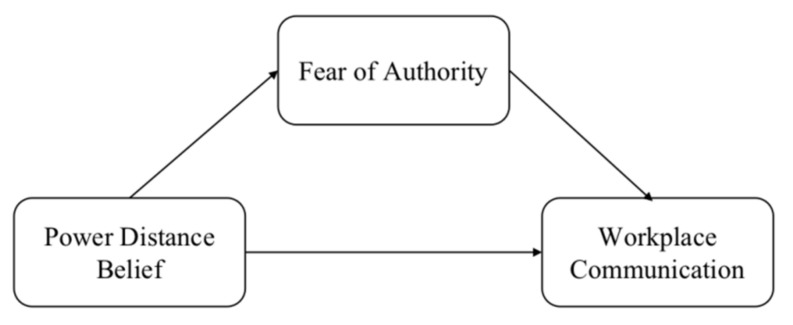
A mediation model of power distance belief, fear of authority, and workplace communication.

To summarize, empirical research is needed to explore the effect of power distance belief on workplace communication. How power distance belief impacts workplace communication requires further investigation to buffer the negative impacts. We hypothesize that individuals with higher power distance belief experience more fear of authority, which negatively affects communication (Figure 1). We will test these hypotheses through four studies.

### 1.3. The Current Research

We focused, in four studies, on the correlational and causal relation between power distance belief and workplace communication as well as the mechanisms under this relation. In Study 1, we examined the association between individual differences in power distance belief and workplace communication. In Study 2, we tested whether fear of authority mediates the relationship between power distance belief and workplace communication via a questionnaire study in three stages over a three-month period. In Study 3, we conducted an empirical experiment to re-test the mechanisms and provided causal evidence. We manipulated power distance belief and assessed fear of authority as well as workplace communication. In Study 4, we conducted a cross-cultural study, recruiting Chinese participants and American participants, to further test the hypothesis and improve the robustness. We recruited participants from high power distance belief culture (i.e., China) and low power distance belief culture (i.e., the United States) and assessed fear of authority as well as workplace communication. Due to COVID-19, we recruited participants on online recruitment platforms (i.e., Credamo, Turkprime) which are reliable and have been widely used [54,55]. Meanwhile, we used questions to exclude participants who do not have workplace communication experience. In addition, we recruited different samples for the four studies to improve the robustness. We expected that people with high power distance belief would have lower communication efficiency in the workplace and that fear of authority would account for the inefficient communication. The study was conducted according to the guidelines of the Declaration of Helsinki and approved by the Ethics Committee of Peking University.

## 2. Study 1

In Study 1, we conducted a correlational investigation and tested H1 at dispositional level: power distance belief is negatively associated with workplace communication. To attain a nuanced understanding of power distance belief’s effect on communication, we examined three attitude outcomes—communication with superiors, communication with colleagues, and communication with subordinates.

### 2.1. Method

#### 2.1.1. Participants

Based on Monte Carlo simulations, Schönbrodt and Perugini [56] recommended using a sample approaching 250 for stable estimates of bivariate correlations. We recruited 250 Chinese participants on the online platform Credamo [57]. Credamo, the Chinese equivalent of Mturk, is a reliable Chinese recruitment platform [54,55]. We excluded 4 for failing an attention check, leaving 246 participants (246 Han Chinese, 0 other; 127 women, 119 men; *M*_age_ = 25.88, *SD*_age_ = 5.37) in the sample. Of them, 8.5% had a high school or college degree, 79.7% had a bachelor’s degree, and 11.8% had a master’s or PhD degree.

#### 2.1.2. Procedure and Materials

We used a question to excluded participants who had never experienced workplace communication before: I have had experience communicating with superiors, subordinates, and colleagues in the workplace. Only participants who answered yes would participate in the survey. This question was shown first in this study and the following studies. We used Brislin’s [58] procedures to translate and back translate all materials and measures from English to Chinese. We measured dispositional power distance belief with the six-item Power Distance Scale [59]. Sample items include “Managers should not delegate important tasks to employees” and “Managers should avoid off-the-job social contacts with employees” (1 = strongly disagree, 5 = strongly agree; *M* = 2.85, *SD* = 0.87, α = 0.80).

We measured workplace communication in three aspects with fifteen self-composed items: communication with superiors, communication with colleagues, and communication with subordinates. We designed the measurement by modifying the communication openness scale [14,60]. We measured information sending and information receiving behaviors, the communication atmosphere, and the contribution to the work task. We measured communication with superiors using five self-composed items. Items are “At work, I can communicate effectively with leaders”, “At work, when I report to the leaders on a project, the leader can clearly understand the project”, “At work, the content of my expression is understandable for the superiors”, “I feel anxiety when I communicate with my leaders”, and “At work, I can accurately understand the meaning of my leaders’ words.” We reversed scored, as appropriate, and averaged responses to create an index of communication with superiors (1 = not at all, 5 = extremely; *M* = 3.71, *SD* = 0.86, α = 0.84). We measured communication with subordinates using five self-composed items. Sample items are “When I communicate with subordinates, she/he can clearly understand what I tell them to do” and “At work, I have misunderstandings with my subordinates because of poor communication.” We reversed scored, as appropriate, and averaged responses to create an index of communication with subordinates (1 = not at all, 5 = extremely; *M* = 3.86, *SD* = 0.58, α = 0.73). (Specific items of communication with subordinates can be found in the Supplementary Materials). We measured communication with colleagues using five self-composed items. Sample items are “At work, I can communicate effectively with colleagues” and “At work, the content of my expression is understandable for the colleagues.” We reversed scored, as appropriate, and averaged responses to create an index of communication with colleagues (1 = not at all, 5 = extremely; *M* = 2.64, *SD* = 0.99, α = 0.76). (Specific items of communication with colleagues could be found in the Supplementary Materials). Workplace communication was measured using 15 self-composed items, including communication with superiors, communication with colleagues, and communication with subordinates. We reversed scored, as appropriate, and averaged responses to create an index of workplace communication (1 = not at all, 5 = extremely; *M* = 3.40, *SD* = 0.49, α = 0.79). Answers to demographic questions concluded the session.

### 2.2. Results and Discussion

As hypothesized, dispositional power distance belief was negatively associated with workplace communication, *r*(246) = −0.31, *p* < 0.001; dispositional power distance belief was negatively associated with communication with superiors, *r*(246) = −0.32, *p* < 0.001; dispositional power distance belief was close to being statistically associated with communication with colleagues, *r*(246) = −0.12, *p* = 0.059; and dispositional power distance was not associated with communication with subordinates, *r*(246) = −0.11, *p =* 0.09.

Consistent with H1, individuals with higher power distance belief exhibit lower communication efficiency in the workplace. Specifically, individuals with high power distance belief communicate with their superiors less often. Meanwhile, power distance belief is not significantly related to communication with subordinates and colleagues. In conclusion, power distance belief hindered only communication with superiors.

## 3. Study 2

Study 1 found a negative relationship between power distance and workplace communication with superiors. In Study 2, we extended our thinking to a mechanism and first tested H2, H3, as well as H4 at a dispositional level. We aimed to test whether fear of authority could account for the negative effect of power distance belief on workplace communication. Here, and in all further studies, we retested H1–H4.

### 3.1. Method

#### 3.1.1. Participants

We used Monte Carlo power analysis for indirect effects [61] (https://schoemanna.shinyapps.io/mc_power_med/, accessed on 5 August 2021) to determine the sample size for our proposed mediational model. We needed at least 163 participants to reach a power of 0.80, assuming correlations of r = 0.30 (SD = 1.00) among the predictor (independent variable), the mediator, and the dependent variable. In anticipation of attrition, we recruited 220 Chinese participants on the reliable online platform Credamo [57]. We excluded 9 for failing an attention check, leaving 211 participants (211 Han Chinese, 0 other; 107 women, 104 men; *M*_age_ = 36.44, *SD*_age_ = 5.26) in the sample. Of them, 8.1% had a high school or college degree, 75.8% had a bachelor’s degree, and 16.1% had a master’s or PhD degree.

#### 3.1.2. Procedure and Materials

We collected data in three stages over a three-month period, using email addresses as the basis for matching and tracking. We measured dispositional power distance belief with the six-item Power Distance Scale [59], as in Study 1 (1 = strongly disagree, 5 = strongly agree; *M* = 2.85, *SD* = 0.88, α = 0.80). After six weeks, fear of authority was measured by the eight-item Fear of Authority Scale [30]. Sample items are “I feel fear in front of people with higher ranks” and “I feel uncomfortable when I interact with people of high status” (1 = not at all, 5 = extremely; *M* = 3.41, *SD* = 0.76, α = 0.84). Six weeks later, we measured workplace communication with superiors, as in Study 1 (1 = not at all, 5 = extremely; *M* = 3.79, *SD* = 0.65, α = 0.75). Answers to demographic questions concluded the session.

### 3.2. Results and Discussion

#### 3.2.1. Correlations

As in Study 1 and consistent with H1, dispositional power distance belief was negatively associated with workplace communication with superiors, *r*(211) = −0.21, *p* = 0.003. Consistent with H2, fear of authority was negatively associated with communication with superiors, *r*(211) = −0.34, *p* < 0.001. Consistent with H3, dispositional power distance belief was positively associated with fear of authority, *r*(211) = 0.56, *p* < 0.001.

#### 3.2.2. Mediational Analysis

**Figure 2:**
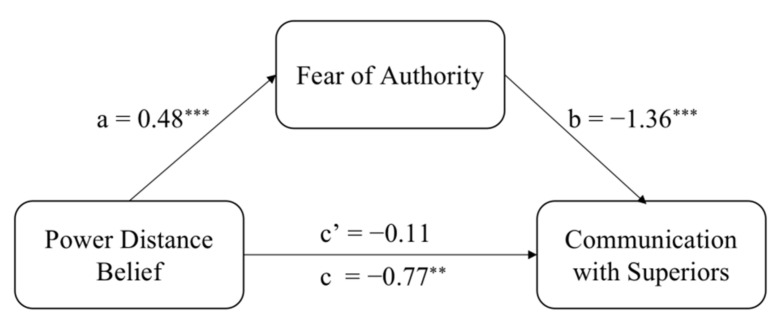
Fear of authority mediates the relationship between power distance belief and communication with superiors in Study 2. Unstandardized coefficients displayed. ** *p* < 0.01, *** *p* < 0.001.

We carried out a bootstrapping mediational analysis [62] (PROCESS, model 4) with 5000 iterations. We entered dispositional power distance belief as an independent variable, fear of authority as a mediator, and workplace communication with superiors as a dependent variable. The indirect effect was significant, *b* = −0.18, *SE* = 0.05, 95% (−0.2840, −0.0794) (Figure 2). Controlling for age and education, the indirect effect remained significant, *b* = −0.20, *SE* = 0.06, 95% (−0.3120, −0.0920). Fear of authority mediated the relation of dispositional power distance belief on workplace communication with superiors, in support of H4.

## 4. Study 3

In Study 3, we aimed to extend Study 2 in two ways. First, Study 1 and Study 2 provided correlational evidence for the hypotheses. In Study 3, we conducted an experimental study and provided further causal evidence. We manipulated power distance belief with the sentence-scrambling task [63], as in former studies [64,65]. In the sentence-scrambling task, participants construct sentences by only using provided words. The completed sentences are related to social hierarchy or equality. When participants complete ten sentences and focus on sentences associated with the power-distance belief, the power-distance belief should become momentarily accessible [63]. Second, we retested the hypothesis with a different measure of workplace communication, scenario-based decision making. We designed a scenario related to workplace communication and participants needed to make a specific choice [64,66]. We used the choice index to indicate the communication effectivity with their supervisors. Study 4 could further provide robust evidence for the hypotheses.

### 4.1. Method

#### 4.1.1. Participants

According to G*Power analysis [67], at least 172 participants were needed to detect a medium effect size (*f* = 0.25) for a between-subjects design with power 0.90 (α = 0.05).

Additionally, we put an item at the beginning, “Do you have subordinates in workplace’’, and only people who have subordinates in the workplace were allowed to participate in this questionnaire. In anticipation of attrition, we recruited 195 Chinese participants on the reliable online platform Credamo [57]. We excluded 10 for failing an attention check, leaving 185 participants (185 Han Chinese, 0 other; 127 women, 58 men; *M*_age_ = 29.05, *SD*_age_ = 6.58) in the sample. Of them, 15.1% had a high school or college degree, 72.4% a bachelor’s degree, and 12.4% a master’s or PhD degree. We randomly assigned participants to the high power distance (*n* = 93) or low power distance (*n* = 92) condition.

#### 4.1.2. Procedure and Materials

We manipulated power distance belief by the sentence-scrambling task [63,64]. Participants formed meaningful sentences from sets of scrambled words and only used the words being offered. These sentences were related to social hierarchy in the high power distance condition or related to equality in the low power distance condition. Specifically, we presented participants in the high power distance belief condition with ten tasks that express high power distance belief. A sample item is “Necessary superiors our social order obedience from is subordinates to for.” Participants should complete the task by forming these words into “Obedience from subordinates to superiors is necessary for our social order.” We presented participants in the low power distance condition with ten tasks that express low power distance belief. A sample item is “an organization is position in Everyone’s equal.” Participants should complete the task by forming these words into “Everyone’s position in an organization is equal.” We measured state dispositional power distance with the six-item Culture Attitudes Inventor as a manipulation check [59] as in Study 1(1 = strongly disagree, 5 = strongly agree; *M* = 2.30, *SD* = 0.67, α = 0.75).

Fear of authority was measured by the eight-item Fear of Authority Scale [30] as in Study 2 (1 = not at all, 5 = extremely; *M* = 3.34, *SD* = 0.78, α = 0.85).

Subsequently, participants were presented with an imaginary workplace situation that read as follows: “You are an engineer in charge of architectural design. Your supervisor asks you to modify the construction design of a building. His reason is that he wants to compress the cost of construction. And your analysis showed that the current design is the best balance of cost and safety. According to your professional knowledge and analysis, the modified design plan would reduce safety and security but not so much as to cause a safety accident.” Participants had to imagine they were the protagonist of the situation and indicate which of the communication decisions they would make. Participants indicated the extent to which they would make a case for the former plan and the extent to which they would follow the supervisor without communication (reversed item) on a seven-point Likert scale (1 = strongly disagree; 7 = strongly agree; *r* = 0.65, *p* < 0.001). The second item was reversed and the average of their scores reflected the workplace communication (*M* = 5.44, *SD* = 1.22). Higher scores indicated more effective workplace communication. Answers to demographic questions concluded the session.

### 4.2. Results and Discussion

#### 4.2.1. Power Distance Manipulation Check

Participants in the high power distance condition (*M* = 2.49, *SD* = 0.70) felt more power distance belief than those in the low power distance condition (*M* = 2.11, *SD* = 0.58), *F*(1, 183) = 16.08, *p* < 0.001, *η_p_*^2^ = 0.081. The manipulation was effective.

#### 4.2.2. Fear of Authority

As we expected, participants in high power distance condition (*M* = 3.51, *SD* = 0.71) felt more fear of authority than those in the low power distance condition (*M* = 3.17, *SD* = 0.82), *F*(1, 183) = 8.69, *p* = 0.004, *η_p_*^2^ = 0.045, indicating that power distance belief increases fear of authority.

#### 4.2.3. Workplace Communication with Superiors

Participants in high power distance condition (*M* = 5.17, *SD* = 1.40) reported less communication than those in the low power distance condition (*M* = 5.71, *SD* = 0.93), *F*(1, 183) = 9.69, *p* = 0.002, *η_p_*^2^ = 0.050. High power distance belief led to less workplace communication.

#### 4.2.4. Mediational Analysis

**Figure 3:**
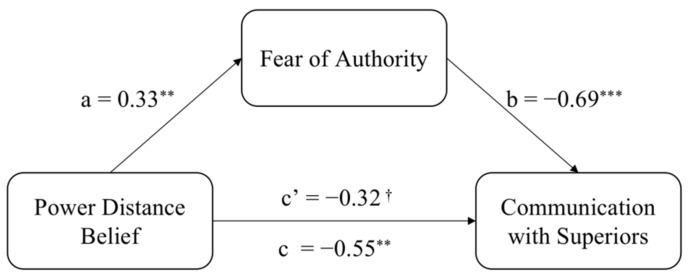
Fear of authority mediates the effect of power distance belief on communication with superiors in Study 3. Unstandardized coefficients displayed. ^†^ *p* < 0.08, ** *p* < 0.01, *** *p* < 0.001.

We carried out a bootstrapping mediational analysis [62] (PROCESS, model 4) with 5000 iterations. We entered the power distance as independent variable (0 = low power distance, 1 = high power distance), fear of authority as mediator, and workplace communication with superiors as dependent variable. The indirect effect was significant *b* = −0.23, *SE* = 0.09, 95% (−0.4168, −0.0740) (Figure 3). Fear of authority mediated the effect of power distance on workplace communication with superiors.

## 5. Study 4

To further test the hypotheses of whether fear of authority mediates the relationship between power distance belief and workplace communication, we conducted a cross-cultural study in Study 4. We manipulated power distance belief by collecting participants with an American or Chinese cultural background, as these cultures exhibit significant differences in power distance belief [7]. According to the hypotheses, Chinese participants with high authority fear would be less effective in communicating with their superiors in the workplace than the American participants.

### 5.1. Method

#### 5.1.1. Participants

A G*Power analysis [67] showed that at least 256 participants were needed to detect a medium effect size (*η_p_*^2^ = 0.03) for a between-subject design with a power of 0.80 (α = 0.05).

In case of any necessary data exclusion, we aimed to recruit 220 American participants (recruitment criteria: residency in the United States, human intelligence test approval rate > 90%) via the Cloud Research Service of Turkprime and 220 Chinese participants on the reliable online platform Credamo [57]. A total of 440 participants participated in Study 4. We excluded 3 participants for noncompletion and 16 for failing attention check questions, resulting in 421 total participants (186 men, 235 women, M_age_ = 34.54, SD_age_ = 7.91), 211 American participants (89 men, 122 women, M_age_ = 38.27, SD_age_ = 8.46) and 210 Chinese participants (97 men, 113 women, M_age_ = 30.70, SD_age_ = 4.99). Among the American participants, 72.5% were White/Caucasian, 10.9% were African American, 5.7% were Hispanic, 8.5% were Asian, 0.9% were Native American, and 1.4% were of other races. For the highest level of education, 7.1% held a senior high school diploma, 28.4% held a college degree, 41.7% held a bachelor degree, 16.6% held a master’s degree, 3.8% held a PhD, and 2.4% held other education certifications. Among the Chinese participants, 100% were Han Chinese. For the highest level of education, 13.9% had a high school or associate’s degree, 75.7% had a bachelor’s degree, and 10.5% had a master’s or PhD degree.

#### 5.1.2. Procedure and Materials

We measured dispositional power distance belief with the six-item Power Distance Scale [59] as in Study 1 (1 = strongly disagree, 5 = strongly agree; *M* = 2.88, *SD* = 0.89, α = 0.80). Fear of authority was measured by the eight-item Fear of Authority Scale [30] as in Study 2 (1 = not at all, 5 = extremely; *M* = 3.06, *SD* = 0.76, α = 0.82). We measured workplace communication with superiors as in Study 1 (1 = not at all, 5 = extremely; *M* = 2.99, *SD* = 1.09, α = 0.79). Answers to demographic questions concluded the session.

### 5.2. Results and Discussion

#### 5.2.1. Power Distance Check

As intended, the Chinese participants (*M* = 3.42, *SD* = 0.73) reported higher power distance scores than the American participants (*M* = 2.35, *SD* = 0.70), *F*(1, 419) = 237.99, *p* < 0.001, *η_p_^2^* = 0.362, and the Chinese participants were more likely to agree with a higher power distance.

#### 5.2.2. Fear of Authority

As we expected, the Chinese participants (*M* = 3.37, *SD* = 0.71) felt more fear of authority than the American participants (*M* = 2.74, *SD* = 0.66), *F*(1, 419) = 90.38, *p* < 0.001, *η_p_*^2^ = 0.177, indicating that power distance increases fear of authority.

#### 5.2.3. Workplace Communication with Superiors

The Chinese participants (*M* = 2.12, *SD* = 0.76) reported less communication than the American participants (*M* = 3.85, *SD* = 0.59), *F*(1, 419) = 702.42, *p* < 0.001, *η_p_*^2^ = 0.626. Individuals with high power distance belief communicated less in the workplace.

#### 5.2.4. Mediational Analysis

**Figure 4:**
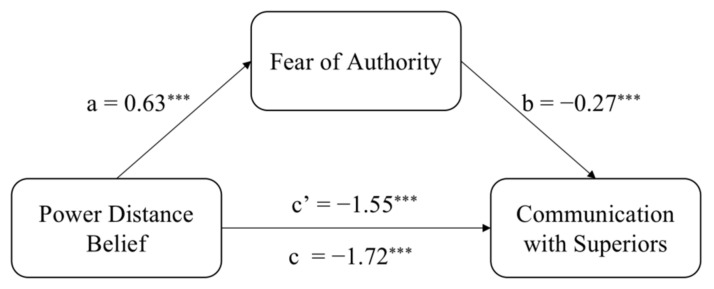
Fear of authority mediates the effect of power distance belief on communication with superiors in Study 4. Unstandardized coefficients displayed. *** *p* < 0.001.

We carried out a bootstrapping mediational analysis [62] (PROCESS, model 4) with 5000 iterations. We entered cross-cultural power distance belief as an independent variable (1 = Chinese participants, 0 = American participants), fear of authority as a mediator, and workplace communication with superiors as a dependent variable. The indirect effect was significant *b* = −0.17, *SE* = 0.03, 95% (−0.2416, −0.1135) (Figure 4). Fear of authority mediated the effect of power distance in different cultures on workplace communication with superiors.

## 6. Discussion

We conducted four studies and verified that high power distance belief negatively impacts workplace communication. We conducted two correlation studies, an experimental study, and a cross-cultural study, providing correlational and causal evidence. Specifically, our findings indicated that high power distance belief hinders workplace communication via less communication with superiors and no variation in communication with colleagues as well as subordinates. Meanwhile, we further uncovered that fear of authority mediated the negative effects of high power distance belief on inefficient communication with superiors.

### 6.1. Research Implications

People with high power distance belief are more inclined to view superiors as authorities. Both subordinates and superiors in high power distance societies are more likely to rationalize inequalities [7,68] of ability, merit, or resources. When people tend to believe that their superiors are better than themselves, they naturally regard them as authorities, which evokes a fear of authority in high power distance societies.

Individuals with higher levels of authority fear communicate with superiors ineffectively, keeping silence and expressing less defiance. Consistent with our findings, studies on emotions found that fear could induce compliance with unreasonable suggestions and elicit stress [69]. This indicates another perspective, that people may tend to be silent in the face of superiors out of self-protection. Previous studies indicated that defensive silence is a fear based form of self-protection, involving withholding relevant ideas, information, or opinions [70]. Adherence to appropriate social norms in communicating with authorities is a sign of social adaptability, which allows these subordinates to better survive in the workplace and avoid workplace bullying [71]. Fear of authority exacerbates silent communication.

It is important to buffer the negative effect of high power distance belief on communication in organizations and societies. Our findings indicated the negative impact of high power distance on workplace communication. Previous studies proposed that fear and power distance belief also changed the original rational intentions of team members [72]. Based on our findings, we proposed three suggestions to reduce the negative effects of high power distance belief on workplace communication. First, reducing the power distance between the superiors and subordinates could contribute to workplace communication. Specifically, the shortened physical distance could decrease the sense of power distance [73]. Similarly, students with high power distance belief improved communication when their teachers maintained a low power distance relationship with them [6]. Policies reducing actual interpersonal distance may improve the inefficient communication associated with high power distance belief. Second, superiors could reduce subordinates’ fear of authority to enhance effective communication. Specifically, more impartial behavior by supervisors could reduce subordinates’ stress and eliminate subordinates’ nervousness when confronted with their leaders [74]. A servant leadership style reduces subordinates’ fear of their direct supervisors [75]. In contrast, abusive supervision increases subordinates’ fear [76]. Supervisors’ behavior and leadership style contribute to better communication by eliminating subordinates’ fear. Third, subordinates could reduce the fear of authority actively. People with a critical thinking style are more likely to take a holistic view of issues and buffer the fear of authority [77]. In addition, social connectedness increases the sense of perceived social support and thus decreases fear and improves communication with others [78]. Our research reveals the relationships and mechanisms between power distance belief and workplace communication. Effective communication further enhances productivity [79] and prevents workplace accidents [80,81].

### 6.2. Limitations and Future Directions

First, in Studies 1, 2 and 4, we measured workplace communication through a self-developed scale. Although we addressed this limitation by testing the reliability and validity of the scale, we should be cautious about our findings regarding workplace communication. Future research should further explore diverse and valid measures of workplace communication.

Second, in current research, we found that fear of authority mediated the effect of power distance belief on workplace communication. As discussed, we proposed shortened power distance, supervisor’ behavior, and subordinates’ thinking style as potential factors that could be used to buffer the undesirable resultant effects. Future research could also test the inhibitory solutions and provide experimental evidence.

Third, in the current study, we recruited participants via an online platform. Although online recruitment platforms (i.e., Credamo; Turkprime) are reliable and have been widely used [54,55] and we also used questions to exclude participants who do not have workplace communication experience, nevertheless, longitudinal design and field study would be needed to complement our experimental and cross-sectional work. Future research could further conduct field studies (i.e., data collection in companies) and explore diverse measures (i.e., evaluate communication by other-reported) to further provide more cogent evidence.

## 7. Conclusions

In conclusion, we found that high power distance belief hinders workplace communication via less communication with superiors due to fear of authority. In addition to objective conditions and technology, subjective factors such as power distance belief are worthy of attention. Fear of authority is another important aspect for organization managers to consider when improving workplace communication.

## Data Availability

The data presented in this study are available on request from the corresponding author.

## Supplementary Materials

The following are available online at https://www.mdpi.com/article/10.3390/ijerph19052932/s1.
