# Supplementary material for: Power Distance Belief and Workplace Communication: The Mediating Role of Fear of Authority
Source: Int J Environ Res Public Health. 2022 Mar 2;19(5):2932. doi: 10.3390/ijerph19052932 (PMC8910159; doi:10.3390/ijerph19052932)
Supplement: Supplementary file 1 [file ijerph-19-02932-s001.zip › ijerph-1536845-supplementary.pdf]

SUPPLEMENTARY MATERIALS

Power Distance Belief and Workplace Accidents - the Mediating Role of Fear of Authority

Study 1 (Correlational)

Power Distance Scale

Please respond to the following statements based on how you feel about the relationship between managers and subordinates. （1 = *Strongly disagree*, 5 = *Strongly agree*）

1. Managers should make most decisions without consulting subordinates.
2. It is frequently necessary for a manager to use authority and power when dealing with subordinates.
3. Managers should seldom ask for opinions of employees.
4. Managers should avoid off-the-job social contacts with employees.
5. Managers should not delegate important tasks to employees.
6. Employees should not disagree with management decisions.

Workplace Communication

Please indicate your level of agreement with each of the following items on a 1 to 7 scale. (1 = *not at all*, 7 = *extremely*)

Communication with Superiors

1. At work, I can communicate effectively with leaders.
2. At work, I can accurately understand the meaning of my leaders' words.
3. At work, the content of my expression is understandable for the superiors.
4. At work, when I report to the leaders on my work, the leader can clearly understand the project.
5. *I feel anxiety when I communicate with my leaders.*

Communication with Subordinates

6. When I communicate with subordinates, she/he can clearly understand what I tell them to do.
7. When my subordinates make mistakes or have problems, I communicate with equanimity.

8. At work, I make misunderstandings with my subordinates because of poor communication.
9. *At work, I communicate with subordinates in a commanding tone.*
10. There is a harmonious atmosphere when I communicate with subordinates.

Communication with Colleagues

11. When I communicate with colleagues, she/he can clearly understand what I express.
12. *At work, my colleagues and I often have conflicts.*
13. At work, I provide immediate feedback to colleagues.
14. At work, I can communicate effectively with colleagues.
15. At work, the content of my expression is understandable for the colleagues.

Reverse-phrased items are italicized.

Demographic Information

What is your age?

What is your gender? 1 = male, 2 = female

What is your race?

1 = Han, 2 = Other

Education: What is the highest degree you have received?

1 = Primary School

2 = Junior Middle School

3 = Senior High School

4 = College

5 = Bachelor's

6 = Master's

7 = PhD

8 = Other

Study 2

Power Distance Scale

Same as in Study 1.

Fear of Authority

Please indicate your level of agreement with each of the following items on a 1 to 5 scale. (1 = *not at all*, 5 = *extremely*)

1. I feel fear in front of people with higher ranks.
2. I became very upset when the leaders express evaluation with me.
3. I try not to offend the leaders.
4. I feel uncomfortable when I interact with leaders or other people of high status.
5. I tend to obey those who are in authority.
6. I think people in authority are quite mysterious.
7. When I am talking to authorities, I worry about what they may be thinking about me.
8. I am concerned about authorities' opinions of me.

Communication with Superiors

Same as in Study 1.

Demographic Information

Same as in Study 1.

Study 3 (Experimental)

Power Distance Belief Manipulation

Here is a sentence task. Specifically, please make a sentence using the words given. Note that you must use all the words given in the question, and you cannot add other words.

High Power Distance Belief

1. Social order for is hierarchy our necessary.
2. Necessary superiors our social order obedience from is subordinates to for.
3. A defined place have should everyone high or low.
4. World in this a social hierarchy should be this.
5. Function to properly subordinates is necessary from obedience for society.
6. Is important to maintain order in society a hierarchy.
7. To maintain social order it is important even if power is unequal.
8. Obey professors students must to function properly for a classroom.
9. An organization has a place in everyone even if high or low.

10. Are necessary differences in power to maintain order.

Low Power Distance Belief

1. Social order for is hierarchy our unnecessary.
2. Not necessary superiors our social order obedience from is subordinates to for.
3. Equal everyone created is.
4. World in this a social hierarchy not should be this.
5. Function to properly subordinates is unnecessary from obedience for society.
6. Is unimportant to maintain order in society a hierarchy.
7. Equal to it is maintain important.
8. To obey professors students don't need to function properly for a classroom.
9. Palce in an equal everyone has an organization.
10. In necessary society equality is.

Power Distance Belief Manipulation Check

Please respond to the following statements based on how you feel about the relationship between managers and subordinates. （1 = *Strongly disagree,* 5 = *Strongly agree*）

1. Managers should make most decisions without consulting subordinates.
2. It is frequently necessary for a manager to use authority and power when dealing with subordinates.
3. Managers should seldom ask for opinions of employees.
4. Managers should avoid off-the-job social contacts with employees.
5. Managers should not delegate important tasks to employees.
6. Employees should not disagree with management decisions.

Fear of Authority

Same as in Study 1.

Communication with Superiors

Suppose you are an engineer. Imagine you are an engineer in charge of architectural design. Your supervisor asks you to modify the construction drawings for a building. His reason is that he wants to compress the cost of construction. And your analysis showed that the current design is the best balance of cost and safety. According to your professional knowledge and

analysis, the modified design plan would reduce safety and security but not so much to cause a safety accident. Please imagine the experience as vividly as possible and indicate your level of agreement with each of the following items on a 1 to 7 scale. (1 = *not at all*, 7 = *extremely*)

I would make a case for the formal plan.

I would follow the supervisor without communication.

Demographic Information

Same as in Study 1.

Study 4 (Cross Culture)

Power Distance Scale

Same as in Study 1.

Fear of Authority

Same as in Study 1.

Communication with Superiors

Same as in Study 1.

Demographic Information

Chinese:

For age, gender, education, and race: Same as in Study 1.

American:

For age, gender, and education: Same as in Study 1.

Race:

1 = White/Caucasian

2 = African American

3 = Hispanic

4 = Asian

5 = Native American

6 = Pacific Islander

7 = Other
